# Verbal Learning and Longitudinal Hippocampal Network Connectivity in Temporal Lobe Epilepsy Surgery

**DOI:** 10.3389/fneur.2022.854313

**Published:** 2022-06-21

**Authors:** Jacint Sala-Padro, Ariadna Gifreu-Fraixino, Júlia Miró, Antoni Rodriguez-Fornells, Immaculada Rico, Gerard Plans, Mila Santurino, Mercè Falip, Estela Càmara

**Affiliations:** ^1^Epilepsy Unit, Hospital de Bellvitge, Barcelona, Spain; ^2^Cognition and Brain Plasticity Group, Bellvitge Biomedical Research Institute (IDIBELL), L'Hospitalet de Llobregat, Barcelona, Spain; ^3^Department of Cognition, Development and Educational Science, L'Hospitalet de Llobregat, University of Barcelona, Barcelona, Spain; ^4^Catalan Institution for Research and Advanced Studies, ICREA, Barcelona, Spain

**Keywords:** resting-sate fMRI, verbal learning, temporal lobe epilepsy, DMN (default mode network), dorsal attention network (DAN)

## Abstract

**Introduction:**

Learning new verbal information can be impaired in 20–40% of patients after mesial temporal lobe resection. In recent years, understanding epilepsy as a brain network disease, and investigating the relationship between large-scale resting networks and cognition has led to several advances. Aligned studies suggest that it is the integrity of the hippocampal connectivity with these large-scale networks what is relevant for cognition, with evidence showing a functional and structural heterogeneity along the long axis hippocampus bilaterally.

**Objective:**

Our aim is to examine whether pre-operative resting-state connectivity along the long hippocampal axis is associated with verbal learning decline after anterior temporal lobe resection.

**Methods:**

Thirty-one patients with epilepsy who underwent an anterior temporal lobe resection were pre-surgically scanned at 3-tesla, and pre/post-surgery evaluated for learning deficits using the Rey Auditory Verbal Learning Task (RAVLT). Eighteen controls matched by age, gender and handedness were also scanned and evaluated with the RAVLT. We studied the functional connectivity along the (anterior/posterior) long axis hippocampal subregions and resting-state functionally-defined brain networks involved in learning [executive (EXE), dorsal attention (DAN) and default-mode (DMN) networks]. Functional connectivity differences between the two groups of patients (learning intact or with learning decline) and controls were investigated with MANOVA and discriminant analysis.

**Results:**

There were significant differences in the pattern of hippocampal connectivity among the groups. Regarding the anterior connectivity hippocampal pattern, our data showed an increase of connectivity in the pathological side with the DAN (*p* = 0.011) and the EXE (*p* = 0.008) when comparing learning-decline vs. learning-intact patients. Moreover, the non-pathological side showed an increase in the anterior connectivity pattern with the DAN (*p* = 0.027) between learning-decline vs. learning-intact patients. In contrast, the posterior hippocampus showed a reduction of connectivity in the learning-decline patients with the DMN, both in the pathological (*p* = 0.004) and the non-pathological sides (*p* = 0.036). Finally, the discriminant analysis based on the pre-operative connectivity pattern significantly differentiated the learning-decline patients from the other groups (*p* = 0.019).

**Conclusion:**

Our findings reveal bilateral connectivity disruptions along the longitudinal axis of the hippocampi with resting-state networks, which could be key to identify those patients at risk of verbal learning decline after epilepsy surgery.

## Introduction

Temporal lobe surgery is an effective treatment in drug-resistant seizures in temporal lobe epilepsy (TLE) (1). However, patients are at risk of verbal learning impairment; assessed through the Rey Auditory Verbal Learning Task (RAVLT), up to 20–40% of patients can be impaired, as the resection affects mostly the anterior part of the mesial temporal lobe, including the hippocampus, which is fundamental in memory and learning (2).

The hemispherical side of the surgery, the resection extent, the verbal learning skills prior to surgery, or the age of seizure's onset are factors modulating the risk of suffering this decline (3). However, not all patients with epilepsy show verbal learning deficits postoperatively, in that some patients perform similarly before and after surgery (4). Such findings indicate that some patients have the neuroplastic potential to compensate for the resected area, leading to post-operative preservation.

Different neural mechanisms might explain the individual differences in the maintenance of verbal learning despite seizure-related and resection-related damage. Evidence suggests that these mechanisms may lead to changes in the connectivity pattern of different neural networks to cope with the impact of pathology and epileptic seizures. In this sense, disruption of neural networks in TLE patients have been reported to be related with cognitive functions (5). More specifically, a decrease in functional connectivity between the hippocampus and the posterior cingulate cortex (PCC), a key hub in the Default Mode Network (DMN) has been consistently reported in relation to verbal memory deficits (5, 6).

Both up-regulation and down-regulation of functional connectivity can be found in patients with TLE (7). Using graph theory measures, a decrease in nodal efficiency of the left hippocampus was related to impairment of verbal memory in patients with left TLE, whereas increases in nodal efficiency of the inferior frontal gyrus and the supplementary motor area were correlated to semantic and phonological fluency in patients with right TLE (8). For those undergoing surgery, down-regulation ipsilateral to the affected side coupled with concurrent contralateral up-regulation of functional connectivity has been associated with a preserved cognitive outcome after the procedure (9). In this vein, higher connectivity of the pathological hippocampus with the PCC was associated with a high risk of post-surgical decline, whereas the connectivity of the contralateral hippocampus to the PCC related to less risk of decline (6). Finally, another study using graph-theory measures (efficiency, integration, and centrality), reported an enhanced integration of the contralateral hippocampus to be predictive of cognitive preservation after surgery, and increased connectivity in the inferior frontal gyrus was related with preserved performance of language tasks (10). In summary, evidence suggest a bilateral reorganization of network connectivity involving both hippocampi and extra hippocampal structures in patients with TLE, that is related to verbal memory and post-surgical decline.

Further evidence also showed relevant changes along the longitudinal axis in the to-be resected hippocampus. A shift in hippocampal activation during an encoding task from the anterior regions toward more post-surgically preserved posterior regions of the to-be resected hippocampus conferred protection against verbal memory impairment after surgery (11). This would suggest some level of reorganization also along the long hippocampal axis associated with decline after resection, yet their underlying neural mechanisms remain poorly understood. In terms of the functional heterogeneity of the anterior and posterior hippocampus and their involvement in cognitive functions, aligned animal and human neuroimaging studies suggest that cognitive domains superimpose according to a functional gradient along the longitudinal axis (12), which also exhibits differences in functional connectivity in TLE patients (13).

Approaching TLE as a brain network disease (14) has been a framework hypothesis that has improved the understanding of seizures, EEG and MRI findings in patients with epilepsy (15). Noteworthy, evidence from recent years indicate that it is the integration of the hippocampi with bilateral, large scale networks what is relevant for cognitive function (16, 17); these large-scale networks can be measured reliably at rest (18), providing a useful tool for connectivity analysis. Network disruption has proven to be the key underlying some neurological deficits (19), and might be a suitable candidate to predict neuropsychological impairment that is less dependent on lesions in TLE (20). Moreover, differences in connectivity and functionality along the longitudinal axis of the hippocampal formation have revealed two systems (21). Despite both systems playing a role in learning and retrieval tasks, it has been hypothesized that they differ in the connectivity with different large-scale networks, with the anterior portion closely connected to the Dorsal Attention (DAN) and Executive Networks (EXE) and related to encoding of external stimuli, while the posterior portion is more closely connected with the DMN and related to memory retrieval and internal sources of information (22). These large-scale networks involve mainly fronto-parietal areas, and their relation to cognitive functions has long been stablished (23, 24).

In the current study, we have investigated the impact of the bilateral functional connectivity along the longitudinal axis of the hippocampus with these large-scale networks and how this could relate to the risk of verbal learning impairment seen in post-surgical epilepsy patients. Of note, the above-mentioned studies in TLE patients evaluated the connectivity of the hippocampus as a whole. However, the longitudinal axis of the hippocampus displays differences in connectivity with large scale networks, and this fact relates to the cognitive functions supported (13, 21, 22, 25). More specifically, we focused on verbal learning abilities that could be impaired after surgery assessed through RAVLT, since previous studies have demonstrated deficits in patients undergoing temporal lobe surgery (2). Verbal learning requires different cognitive processes to be intact in order to be performed correctly. The RAVLT evaluates verbal learning requiring both retrieval (26) and encoding processes, as well as attentional shifting strategies (27) and executive functioning (28). These processes that are involved in verbal learning require the activation of both the anterior and posterior hippocampal formation.

Specifically, we compared the pre-surgical connectivity of both hippocampi between patients who had an impairment in the RAVLT learning domain after surgery with those who had not. We hypothesized that functional connectivity of the anterior and posterior hippocampal formation with DMN, EXE, and DAN differ in patients with verbal learning impairment after mesial temporal resections already before epilepsy surgery. We also analyzed this connectivity for the hippocampus as a whole, to investigate whether the anterior/posterior division provided further information on these patients. This study offers the possibility to identify biomarkers that predict the prognosis of the surgical outcome, which may be key for the pre-surgical planning.

## Methods

### Participants

We included 31 consecutive patients who underwent an anterior temporal lobe resection for epilepsy surgery in the period 2009–2014. All patients were operated by the same neurosurgical team, and all the tissue was analyzed in the neuropathology department of our hospital. We also scanned 18 controls matched by age (T-student = 0.15, *p* = 0.516), gender (*X*^2^ = 0.44, *p* = 0.834) and handedness (*X*^2^ = 0.016, *p* = 0.9).

From the 31 patients, 18 were women (58.1%), with a median age of 49 years old (range 44 years). Sixteen patients (51.6%) had a left mesial temporal lobe resection, and 24 (77.4%) had signs of hippocampal sclerosis on the tissue sample. After surgery, 14 patients (45.2%) remained completely seizure free during follow-up (median time of follow-up 76 months, range 55 months). There was no difference among patient's groups in terms of seizure freedom after surgery (learning decline 45.5% of seizure freedom vs. learning intact 60% of seizure freedom, *X*^2^ = 0.61, *p* = 0.436). See Table 1 for demographic details of the patients and healthy control group samples. The study was conducted in accordance with the principles of the Declaration of Helsinki. Written informed consent was obtained for every participant, and the study was approved by the Clinical Research Ethics Committee of Bellvitge University Hospital.

**Table 1:** Sociodemographic and clinical characteristics of Temporal Lobe Epilepsy patients and controls.

	**Learning decline**	**Learning intact**	**Controls**	***p***
	***N =* 11**	***N =* 20**	***N =* 18**	
Age (years)	47 (7.5)	51.7 (12.1)	49.5 (11.9)	0.516
Sex (female)	6 (54.5%)	12 (60%)	11 (61.1%)	0.937
Age at onset (years)	18.1 (13.3)	12.6 (10.4)		0.207
Left resection (participants)	8 (72.7%)	8 (40%)		0.081
HS (participants)	8 (72.7%)	16 (80%)		0.643
Seizure-free^*^ (participants)	6 (54.5%)	8 (40%)		0.477
Post-surgical follow-up^**^	86 (50)	93.5 (55)		0.919
**Hipp volume (cm** ^**3**^ **)**				
Pathological	2.77 (0.5)	2.8 (0.5)	3.33 (0.3)	**<0.001**
Contralateral (Participants)	3.41 (0.2)	3.52 (0.2)	3.41 (0.3)	0.154
RAVLT pre	12.2 (2.2)	11.1 (2.2)	11.7 (2.4)	0.472
RAVLT post	8.2 (1.2)	11.5 (2.7)	11.7 (2.8)	**0.002**

*Data presented as number (percentage). N, number of participants; HS, Hippocampal Sclerosis; Hipp, Hippocampus; RAVLT, Rey Auditory Verbal learning Test*.

* *At the end of follow-up*.

** *In months, median (range). Significance assessed with the Mann-Whitney test. The bold values indicate the statistically significant*.

#### Verbal Learning Assessment

All patients were assessed using the RAVLT (29) before and after surgery for evaluation of cognitive functioning. The stimuli that were presented in each of the evaluation were different. The test was performed in a median time of 9 months (range 5–19 months) before surgery, while the evaluation after surgery was performed in a median time of 6 months (range 4–12 months). Between both tests the span of time was a median of 17 months (range 11–28 months). In this task, the evaluator reads 15 different words to the patients. In an immediate recall test, patients are asked to repeat after each list all the words they could remember, regardless of the order in which they were presented by the experimenter. This task was repeated five times in a row. The final performance in the five lists was recollected as the absolute number of words remembered in the last run for each subject. This final score as an absolute number of words was used as a measure of verbal learning performance. Then, this score was transformed into standardized values using normative data from Hispanic cohorts, corrected for age, gender and educational level (30).

#### MRI Data Acquisition

Patients underwent a pre-operative whole-brain structural MRI scans using a 3.0 Tesla Siemens Trio MRI. A 32-channel phased-array head coil system was used to acquire high-resolution T1-weighted images (slice thickness = 1 mm; no gap; number of slices = 240; TR = 2,300 ms, TE = 3 ms, matrix = 256 × 256; FOV = 244 mm; voxel size 1 × 1 × 1 mm). Resting state fMRI data were collected using a single-shot T2^*^-weighted gradient-echo EPI sequence (slice thickness = 4 mm; no gap; number of slices = 32, interleaved order; TR = 2,000 ms; TE = 29 ms; flipangle = 80°; matrix = 80 × 80; voxel size = 3 × 3 × 4 mm^3^, 110 volumes). During the resting state, participants were instructed to keep still with the eyes closed but not fall asleep, and to not focus on any thoughts, as far as possible. Healthy participants underwent the same neuroimaging protocol.

#### Hippocampal Volumes

Total hippocampal volumes were segmented from a fully automated pipeline for hippocampal subfields including the automated cortical parcellation and subcortical recon-all tools implemented in FreeSurfer 6.0 (http://surfer.nmr.mgh.harvard.edu/). The technical details have been described previously (31). To adjust for differences in head size, all volumes were normalized to total intracranial volume (ICV) by dividing by intracranial ICV calculated using FreeSurfer. Finally, all generated hippocampal images were visually inspected to ensure there were no technical failures or mislabeling.

#### Resting-State Functional Connectivity Analysis

Independent Component Analysis (ICA) was used to delineate spatially independent and temporally coherent patterns of functional brain connectivity in the resting state DMN, EXE and DAN.

Individual functional data pre-processing was carried out using Statistical Parameter Mapping software (SPM12, Welcome Department of Imaging Neuroscience, University College, London, UK, http://www.fil.ion.ucl.ac.uk/spm/) following the standard protocol. The preprocessing of the data included realignment, co-registration between the structural T1 and their respective mean functional image, normalization, spatial smoothing (FWHM 8 mm). Then, to extract the different functional networks by means of ICA, we used the GIFT software (http://icatb.sourceforge.net/) (32). Thus, smoothed data of all participants, combining patients and healthy controls, were temporally concatenated into a 4D time series and then decomposed into different temporal dimensions using principal component analysis and constrained to 20 components, to be then analyzed with the Infomax algorithm (33).

ICA was performed 100 times and the results were clustered by GIFT toolbox ICASSO min cluster size of 15 and max of 20 (number of runs) and RandInit and Bootstrap were selected. Finally, there was a back-reconstruction process from the group ICA components estimated to the individual activation values for each participant of the different groups and for each component. This process allowed the estimation of different spatial component maps for each individual in terms of voxel-wise *z* scores. Then, within the obtained ICA networks in the group of participants, we identified the common resting-state intrinsic connectivity networks by visual inspection, and among them, we selected the networks of interest. Two independent authors (JS, EC) separately reviewed the 20 components from the ICA, and after visual inspection and in accordance to published data (23) identified the DMN, DAN and EXE networks (see Table 2).

**Table 2:** Resting-state network functional connectivity for controls and patients (*p* < 0.05 FWE-corrected at whole brain level, cluster extent >20 voxels).

**Anatomical region**	**Cluster size**	**MNI coordinates**	***t* value**
	**(voxels)**	**(x y z)**	
**For the default mode network**			
L superior medial frontal	6,803	−4 48 30	32.4
R precuneus		12 −52 18	26.4
L Precuneus		−6 −58 16	25.1
L Angular gyrus	2,378	−42 −72 30	25.0
R Angular gyrus	1,815	48 −62 24	19.9
L orbitofrontal	2,912	0 62 −2	17.6
L medial frontal		2 64 10	15.5
L parahipocampal	88	−24 −38 −14	10.3
R middle frontal gyrus	307	24 34 44	10.3
R superior frontal gyrus		26 24 54	9.1
L superior frontal gyrus	245	−18 40 48	8.5
L middle frontal gyrus		−24 26 50	8.1
L middle temporal gyrus	102	−60 0 −22	8.3
**For the executive network**			
R Supp motor area	11,429	2 12 64	17.8
R Frontal superior medial		10 22 60	17.2
L Frontal superior medial		−6 62 18	16.2
L Superior parietal	76	−8 −58 56	8.6
R Superior parietal		6 −56 54	6.2
R Frontal superior medial	25	18 16 10	7.6
L Caudate	26	−12 0 16	7.2
L middle temporal gyrus		−60 −6 −14	7.6
**For the dorsal attention network**			
R Superior parietal	7,742	12 −66 58	18.0
L Superior parietal		−8 −60 62	15.6
R Middle frontal gyrus	1,937	24 14 58	11.8
L Supp motor area		−2 2 50	11.7
L Mid-cingulate		2 16 38	11.3
L middle frontal gyrus	444	−24 4 58	10.9
L Supramarginal	80	−58 −28 34	8.4
R Posterior cingulate	81	10 −52 8	8.3

*MNI, Montreal Neurological Institute stereotactic space; L, Left, R, right; Supp, Supplementary*.

#### Functional Connectivity of the Hippocampus and the Networks of Interest

For the resting-state fMRI data, the group-level EXE, DAN and DMN maps were identified using a one-sample *t* test (including both positive and negative effects) after entering the individual resting-state maps for both patients and controls. In order to define the group level resting-state connectivity map for each network, results were reported using a threshold of *p* < 0.05 with Family-wise error correction for multiple comparisons at whole brain level. The maxima of suprathreshold regions were localized by rendering them onto a normalized T1 structural MNI reference brain.

Then, for each participant, the whole hippocampi and four seed regions (left/right, anterior/posterior) were defined based on hippocampal delineations and, the z-scores across all voxels within the selected region of interest (ROI) were averaged in the different networks at *p* < 0.05 (uncorrected), representing the functional connectivity magnitude between the hippocampal ROI and each network.

The different ROIs were delineated following the same approach described previously (34). Specifically, the segmentation of hippocampi was performed according to the Anatomical Automatic Labeling (AAL) brain atlas (35). The anterior and posterior subdivision was made just posterior of the uncal apex ranged from y = −2 to −18 (anterior) and from y = −24 to −42 (posterior) in MNI coordinates. Between the anterior and posterior part of the hippocampal division, a gap of 4 mm was left in order to reduce inter-regional effects because of smoothing and registration errors (17).

### Statistical Analysis

The statistical analysis was performed with SPSS (v.25, SPSS Inc., Chicago, USA). First, in order to identify those patients that presented cognitive impairment after surgery, we compared the scores of verbal learning task before and after surgery on an individual level. Specifically, we classified those patients who had a decline of two standard deviation of the normalized score or more in verbal learning as learning-decline, and the patients who showed a decline below two standard deviation or no decline after surgery as learning-intact. In order to define significant decline we applied the two standard deviation cut-off as it is a highly stringent according to previous reports (36).

Second, statistical analysis of groups (learning-decline vs. learning-intact vs. controls) demographics, clinical data including learning scores, and total hippocampal volume was performed. Log linear analysis and ANOVA were used to describe socio-demographic, clinical and hippocampal volume differences. If there was a significant difference among groups, a *post-hoc* univariate analysis using Bonferroni's correction was performed. When comparisons were made between learning-decline and learning-intact groups, T-student and Chi-square tests were performed to assess statistical significance.

Third, to investigate differences in the hippocampal longitudinal axis connectivity according to the neuropsychological performance, a multivariate analysis of variance (MANOVA) with Tukey *post-hoc* test was performed, considering the three groups (controls, learning-decline and learning-intact). First, Box's test was performed to check equality of covariance. Then, overall significance of the MANOVA test was assessed with Pillai's trace. This was followed by univariate analysis with Tukey correction. Finally, in order to corroborate which hippocampal longitudinal axis connectivity with the different networks outcomes could be used to correctly classify the different groups based on the surgical cognitive deficits, a linear discriminant analysis was performed, and a receiver characteristic operator curve (ROC) was configured for the variables showing significant differences on the discriminant function. These analyses were performed for the anterior/posterior division and for the whole hippocampus separately.

## Results

After classifying the patients according to the cognitive deficits postoperatively in the verbal learning task, 20 patients were learning-intact and 11 suffered a learning-decline after surgery. Preoperatively, no differences were found between groups on the RAVLT. After surgery, a significant difference was found when comparing controls and learning-intact patients with the patients of the learning-decline group (*F* = 7.09, *p* = 0.002). See Table 1 for socio-demographic and clinical differences.

Global hippocampal volume differences among the three groups were investigated. Comparing the pre-surgical hippocampal volume, both the learning-intact and learning-decline groups had a decreased hippocampal volume for the to-be resected side when compared with the controls (*F* = 9.79, *p* < 0.001) with no significant differences between the two groups of patients on the *post-hoc* analysis. Also, there were not significant differences in the hippocampal volume among groups on the first-level ANOVA for the contralateral side (*F* = 1.97, *p* = 0.154).

**Figure 1:**
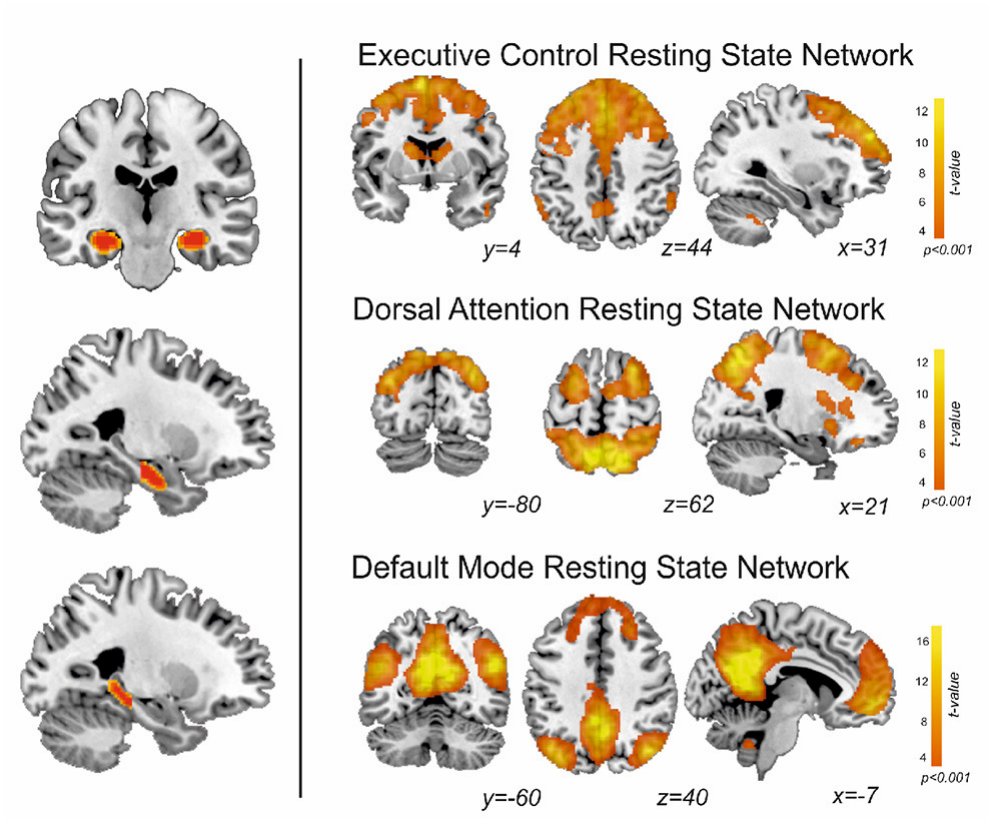
On the left, segmentation of both hippocampi, and division along the anterior-posterior axis. On the right, the resting state networks obtained in this study from patients and controls.

Group-level EXE, DAN and DMN map of patients and controls is shown in Figure 1. Regions with a positive coupling corresponded to areas typically reported in the literature as part of the EXE (i.e., dorsal and anterior frontal areas), DAN (i.e., superior parietal and dorsolateral frontal areas) and DMN (i.e., posterior cingulate-precuneus and orbitofrontal regions). We first performed MANOVA among the three groups (learning decline, learning intact, and controls) comparing the connectivity of the whole hippocampus with the DMN, EXE, and DAN networks. Using Pillai's trace, there was a significant difference of the pattern of hippocampal connectivity among the groups [V = 0.95, *F*_(12,84)_ = 6.3, *p* < 0.001]. See Table 3 for details.

**Table 3:** Connectivity analysis results for each group.

		**L-D**	**L-I**	**Controls**	***p***
**Pathological side**					
Anterior hippocampus	DMN	1.2 (2.6)	0.009 (2.2)	1 (2.1)	n.s.
	DAN	1.56 (2.5)	−0.86 (2.3)	−0.37 (1.8)	**<0.05**
	EXE	2.6 (3.5)	−0.61 (2.6)	1.66 (3.4)	n.s.
Posterior hippocampus	DMN	−1.27 (2.1)	0.44 (1.9)	0.96 (1)	**<0.05**
	DAN	0.57 (2.6)	0.41 (2.3)	0.12 (2.5)	n.s.
	EXE	0.13 (3.2)	−0.64 (2.1)	−1.1 (2.3)	n.s.
Whole hippocampus	DMN	−0.38 (2.08)	−0.86 (1.96)	3.64 (2.1)	**<0.05**
	DAN	0.98 (0.98)	0.13 (1.8)	−1.15 (2)	**<0.05**
	EXE	0.1 (2.02)	−0.094 (1.96)	−0.13 (2.4)	n.s.
**Healthy side**					
Anterior hippocampus	DMN	−0.15 (2.8)	0.29 (2.1)	1.31 (1.8)	**<0.05**
	DAN	2.1 (2.7)	−0.11 (2.2)	1.18 (2.3)	n.s.
	EXE	1.92 (1.8)	0.87 (3.4)	1.4 (3.7)	**<0.05**
Posterior hippocampus	DMN	−0.04 (2.3)	1.61 (1.7)	1 (1.4)	**<0.05**
	DAN	0.74 (2.6)	0.44 (2)	0.81 (2)	n.s.
	EXE	−0.002 (2.7)	−1.05 (2.4)	−0.1 (1.9)	n.s.
Whole hippocampus	DMN	−0.81 (1.09)	0.57 (1.42)	4.84 (2.05)	**<0.05**
	DAN	0.82 (2.54)	0.46 (2.18)	−0.82 (2.05)	n.s.
	EXE	0.71 (2.1)	−0.56 (2.34)	0.12 (1.89)	n.s.

*Data represents the z-scores across all values of the hippocampal subregions averaged in the different networks, presented as mean (standard deviation)*.

*L-D, learning decline; L-I, learning intact; DMN, Default Mode Network; DAN, Dorsal Attention Network; EXE, Executive Network. Significance was assessed with a MANOVA test as described previously. The bold values indicate the statistically significant*.

For this whole hippocampus analysis, there were differences on the DMN connectivity with both the healthy side [*F*_(2,46)_ = 51.2, *p* < 0.001] and the pathological side [*F*_(2,46)_ = 30, *p* < 0.001], and on the DAN connectivity with the pathological hippocampus [*F*_(2,46)_ = 5.54, *p* < 0.007]. The *post-hoc* analysis revealed a significant decrease in global hippocampal connectivity bilaterally with the DMN for patients (both learning-decline and learning intact) when compared to controls (*p* < 0.0001, Tukey corrected). The *post-hoc* analysis on the pathological hippocampus with the DAN connectivity revealed an increase in connectivity for the patients with learning decline compared to controls (*p* = 0.007, Tukey corrected).

**Figure 2:**
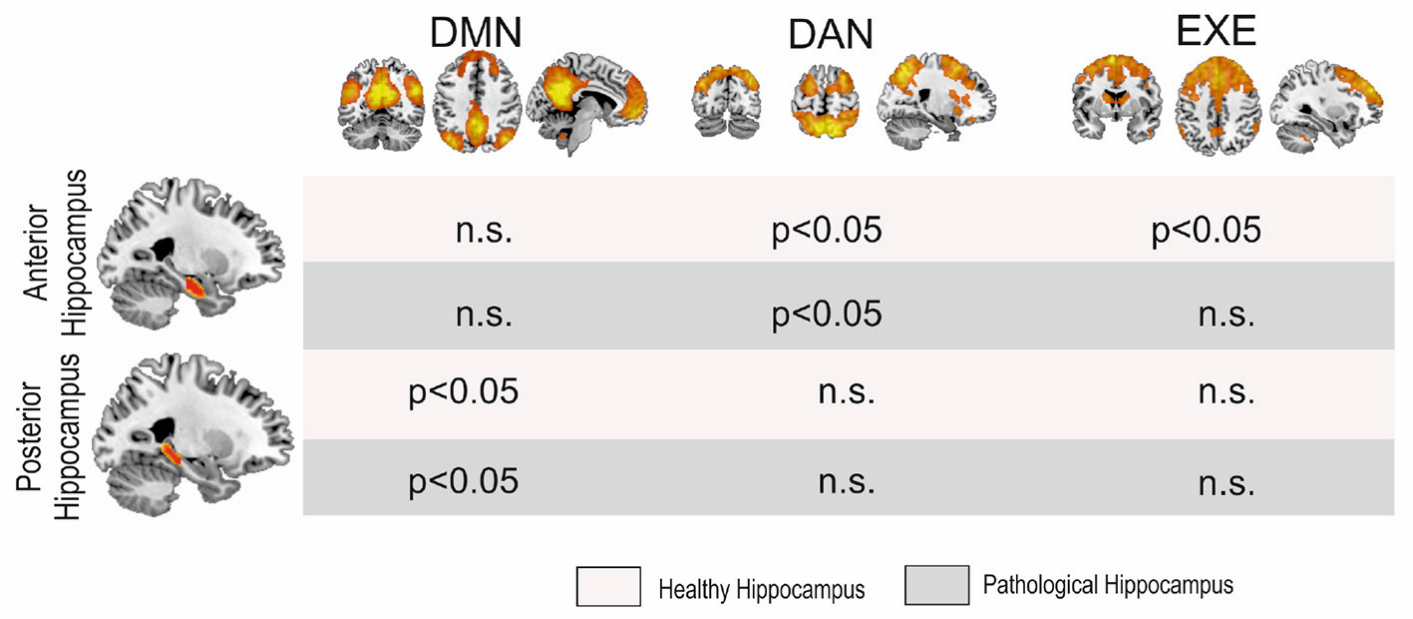
MANOVA results. Significant differences were found among groups in the posterior hippocampi with the DMN and the anterior hippocampi with the DAN and the EXE, although for the latter only with the pathological side. DMN, Default Mode Network; DAN, Dorsal Attentional Network; EXE, Executive Control Network.

We then performed a MANOVA among the three groups comparing the connectivity of the anterior and posterior hippocampi with the DMN, EXE, and DAN networks. Using Pillai's trace, there was a significant difference of the pattern of hippocampal connectivity among the groups [V = 0.73, *F*_(24,72)_ = 1.72, *p* = 0.041]. See Figure 2 for comparison results, and Table 3 for connectivity details.

With regard to the anterior connectivity hippocampal pattern, separate univariate tests revealed a significant difference in the pathological side with the DAN [*F*_(2,46)_ = 4.75, *p* = 0.013] and with the EXE [*F*_(2,46)_ = 5.65, *p* = 0.006]. Posterior *post-hoc* analyses showed that these patterns reflect a significant increase in connectivity in the DAN (*p* = 0.011, Tukey corrected) and in the EXE (*p* = 0.008, Tukey corrected) between learning-decline vs. learning-intact patients. Moreover, the non-pathological side showed a significant difference in the anterior connectivity pattern with the DAN [*F*_(2,46)_ = 3.90, *p* = 0.027], in which further post-doc analyses indicated a significant increase of connectivity (*p* = 0.022, Tukey corrected) between learning-decline vs. learning-intact patients.

**Figure 3:**
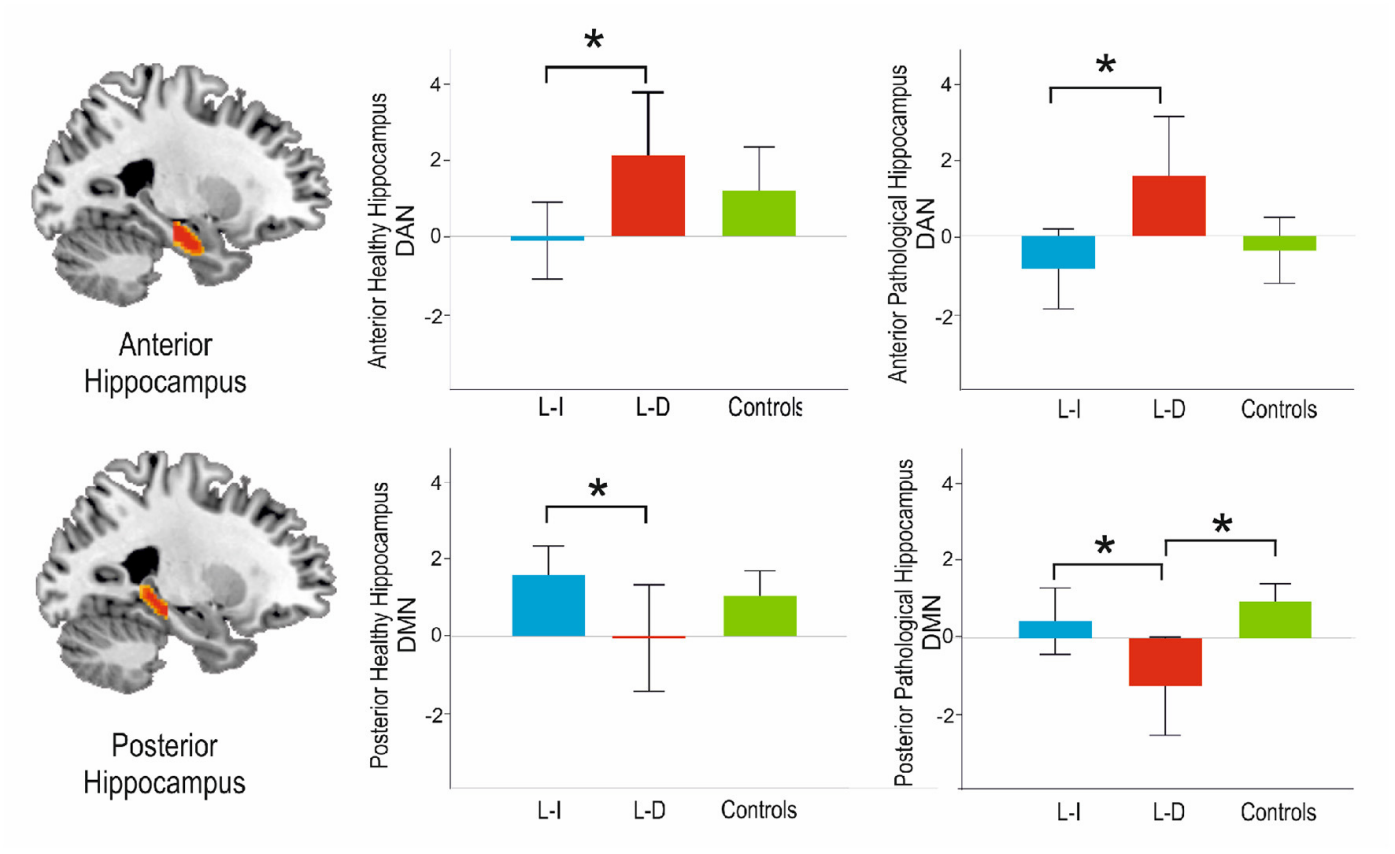
Main results of the *post-hoc* analysis among groups connectivity analyses with the DAN and the DMN along the longitudinal axis of the hippocampus. L-I, Learning Intact; L-D, Learning Decline. *Indicates statistical significance *p* < 0.05.

In contrast, the posterior connectivity hippocampal pattern only showed differences with the DMN, both in the pathological [*F*_(2,46)_ = 6.15, *p* = 0.004], and the non-pathological [*F*_(2,46)_ = 3.57, *p* = 0.036] side. On *post-hoc* analysis, the connectivity of the posterior pathological hippocampus with the DMN was significantly decreased in learning-decline patients when compared with learning-intact patients (*p* = 0.029, Tukey corrected) and controls (*p* = 0.003, Tukey corrected). The connectivity of the posterior non-pathological hippocampus was also significantly decreased in learning-decline patients compared to learning-intact patients (*p* = 0.027, Tukey corrected), but there was no difference with controls. See Figure 3 for results.

The MANOVA was followed up with a linear discriminant analysis, which provided a multivariate model of how the connectivity measures differentiated the groups. As the discriminant analysis was carried out among three different groups, two different functions were obtained, as per differentiate each group. The functions tried to differentiate the three groups of participants considering a minimum weighted combination of the connectivity measures analyzed.

**Figure 4:**
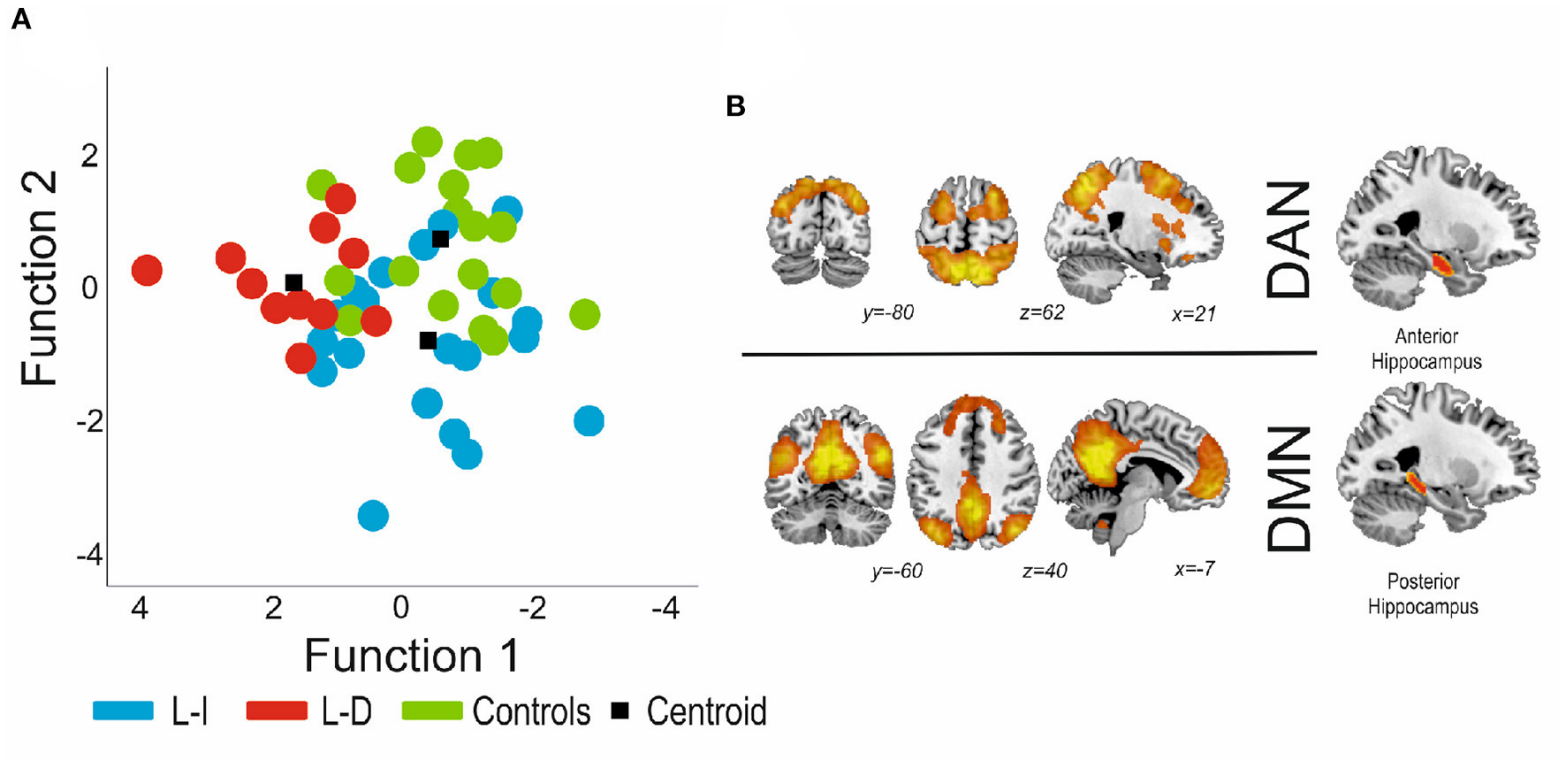
**(A)** Canonical functions distribution of the discriminant analysis. Function 1 was found to significantly distinguish patients with decline from patients without decline and controls. **(B)** The connectivity variables with higher load were both for the pathological hippocampus (posterior part indicates connectivity with the DMN, and the anterior portion indicates connectivity with the DAN). DMN, Default Mode Network; DAN, Dorsal Attention Network.

Using the connectivity of the hippocampus as a whole, the first function significantly discriminated the controls from the patients [Wilkin's lambda = 0.17, *X*^2^ = 77.05, *p* < 0.0001], but the second function was non-significant [Wilkin's lambda = 0.850, *X*^2^ = 7.06, *p* = 0.216], so the model was not capable of appropriately distinguishing the groups of patients, thus not identifying the learning-decline patients. On the other hand, for the anterior/posterior division results, the first discriminant function explained 63.3% of the variance, canonical *R*^2^ = 0.67, and significantly discriminated the learning-decline patients from the other groups [Wilkin's lambda = 0.37, *X*^2^ = 40.41, *p* = 0.019]. Individual discriminant scores for this function correlated significantly with the connectivity between the posterior pathological hippocampus and the DMN (0.67), inversely with the connectivity between the anterior pathological hippocampus and the DAN (−0.63), and finally with the posterior contralateral hippocampus with the DMN (0.47). As such, these three variables demonstrated the most importance in terms of learning-decline group differentiation. The second discriminant function explained the 36.7% remaining variance, canonical *R*^2^ = 0.570, but was not significant (Wilkin's lambda = 0.851, *X*^2^ = 7.03, *p* = 0.218]). See Figure 4 for details.

**Figure 5:**
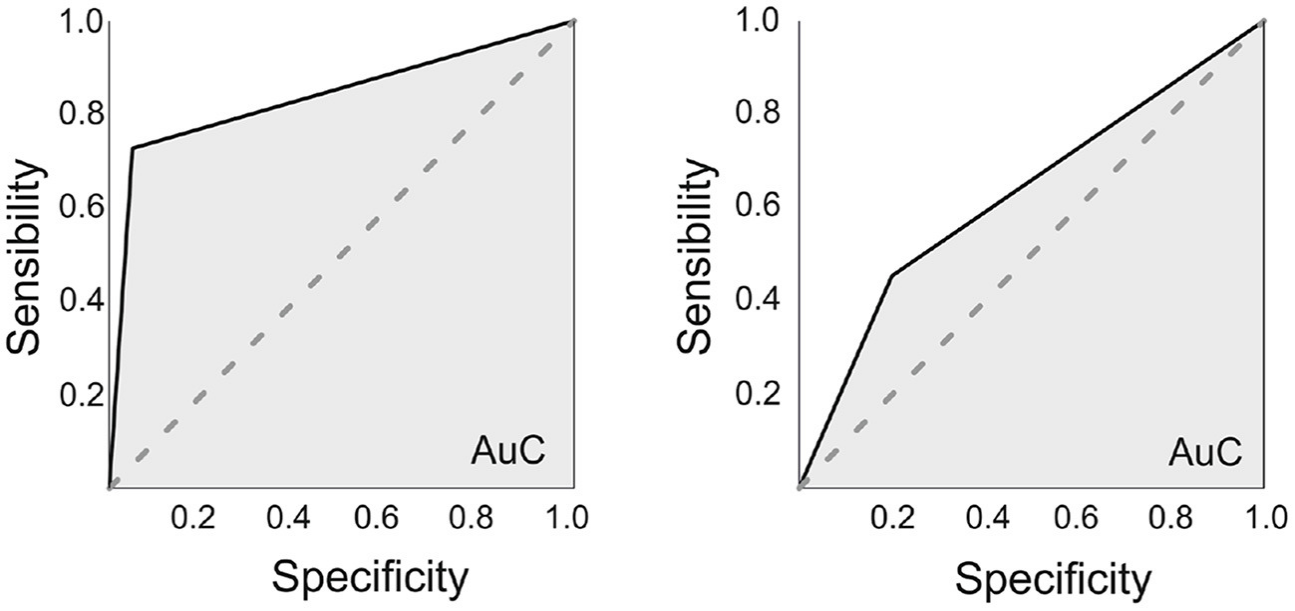
ROC curves. On the left, using the anterior/posterior division of the hippocampi (Connectivity measures for the Posterior bilateral hippocampi-DMN and the anterior to-be resected hippocampus-DAN). The area under the curve (AuC) was of 0.835, with an optimal sensitivity of 0.727 and specificity of 0.95. On the right, using the whole hippocampus (also connectivity measures from the bilateral hippocampi-DMN and the to-be resected hippocampus-DAN). The AuC was 0.63, with an optimal sensitivity of 0.46 and specificity of 0.8.

Finally, a ROC curve was calculated for the optimal cut-off point to predict verbal learning decline from the three previous connectivity variables which showed significance in the discriminant analysis (posterior bilateral hippocampi to DMN and anterior to-be resected hippocampus to DAN). Also, for the connectivity of the whole hippocampus a ROC curve was calculated, using the bilateral DMN connectivity and the pathological hippocampus to DAN connectivity. The results for the hippocampus as a whole, presented an Area under the Curve (AuC) was 0.63, with an optimal sensitivity of 0.45 and specificity of 0.8, whereas for the anterior/posterior connectivity presented an AuC of 0.84, with an optimal sensitivity of 0.73 and specificity of 0.95 (see Figure 5).

## Discussion

In this study, we identified functional connectivity signatures related to post-operative verbal learning decline in TLE patients. That is, different connectivity patterns with the RSN along the longitudinal axis of the hippocampus discriminated among patients with and without severe decline in verbal learning after surgery. In particular, we found a pre-operative increase of connectivity of the anterior to-be resected portion of the hippocampus with the DAN, together with a bilateral decrease of connectivity of its posterior portion with the DMN, that appropriately differentiated the group of patients with post-surgery learning-decline from learning-intact patients and controls. In addition, despite the fact that significant changes were found in connectivity among the anterior hippocampi and the EXE network, these changes did not sufficiently discriminate learning-decline patients according to our model.

Our study revealed two main levels of organization along the long hippocampal axis associated with the verbal learning decline after resection conferring protection against the cognitive impairment after surgery in TLE patients. First of all, according to our data, post-surgery learning-decline patients, learning-intact patients and controls differed in the pattern of the hippocampal formation connectivity with the RSN following an anterior-posterior gradient. Secondly, we found bilateral, up- and down-regulations of resting state connectivity, with significant differences in the learning-decline group vs. the learning-intact and control groups. Finally, in our series, investigating the connectivity along the longitudinal axis proved to discriminate learning decline patients from learning intact patients and controls, as compared with the connectivity of the hippocampus as a whole, which was only capable of discriminating patients from controls.

In recent years, the modular paradigm, i.e., postulating specific brain areas as responsible of complex cognitive tasks, has been shifted toward the study of how neural networks influence cognitive processing (37). According to the network paradigm, cognitive functions arise from distributed brain areas comprising multiple distinct, interacting networks. Several large-scale brain networks have been described using resting state functional imaging. It is this connectivity of these RSN with other brain structures such as the hippocampal formation that has proven to be crucial for the maintenance of cognitive tasks (16, 23).

Verbal learning decline has been a consistent finding after temporal lobe surgery (3). Two models were initially proposed as per why some patients showed verbal memory decline after surgery. Namely, these include functional adequacy of the to-be resected hippocampus as sustaining cognitive function, which is thereby lost upon resection, or instead the functional reserve of the contralateral hippocampus (38). However, more recent work switched the attention from the hippocampal model to the connectivity of widespread neuronal networks in order to explain complex cognitive changes that imply different functions (37). In this regard, aligned task-based functional MRI studies using a combination of language and encoding paradigms showed both hippocampal and extra hippocampal activation differences in identifying patients at risk of decline. Thus, the authors postulated that it was the functional adequacy of the network elucidated in the task what was truly relevant for verbal memory decline (39). Taken together, these results, along with evidence on targeting network dysfunction for neurological symptoms (19), pave the way for analysis beyond the lesional level, exploring the cognitive deficits at a network level. Our main hypothesis builds up from this perspective, exploring the role of the disconnection of the hippocampus with the DMN, DAN and EXE, crucial in verbal learning (16, 17).

Our main findings are in line with this network model. Analyzing resting-state connectivity of the hippocampus with the DMN, DAN, and EXE, bilaterally and along its longitudinal axis, significantly discriminated patients at risk of verbal memory decline; a bilateral decrease of the posterior portion of the hippocampi, together with an increased connectivity of the to-be resected hippocampal anterior portion with the DAN was significantly related to verbal learning decline after surgery. Noteworthy, hippocampal connectivity with the RSN followed a functional gradient, that is, the posterior hippocampus with the DMN and the anterior hippocampus with the DAN. Thus, by segregating the connectivity pattern along the axis of the hippocampus, we found significant differences related to the connectivity of the anterior-posterior system (21, 25).

Previous studies evaluating resting state connectivity also reported differences among patients with and without decline (6, 8, 10). In this sense, the definition of different cognitive networks at rest provides an easy and reproducible framework to assess brain functioning. Network studies at rest in TLE patients have shown widespread alterations and correlations to different neuropsychological tasks (10, 13, 40). Using resting-state connectivity, previous studies showed how the connectivity of the hippocampus and the posterior cingulate cortex (PCC), the key hub in the DMN, differed among patients. Relating to their different verbal memory scores, lower strengths of connectivity between the DMN and the hippocampi correlated with poorer performances (41). Furthermore, changes in DMN-hippocampal connectivity may relate with memory loss after surgery (6). Specifically, evaluating the connectivity of the PCC within the DMN to the hippocampus elucidated how patients with episodic memory loss after surgery showed stronger connectivity with the pathological hippocampus, whereas intact patients showed stronger connectivity with the contralateral hippocampus. In addition, using graph theory measures of areas relevant to cognitive functions and to ictal pathology, Doucet et al. (10) found that neurocognitive deficits after surgery were related mainly to the contralateral hippocampus and widespread bilateral regions, again emphasizing the importance of extratemporal of networks in cognitive functioning.

Nonetheless, the variety of approaches to analyze resting state data, together with the large number of neurocognitive tests reported, limits study comparison. In relation to previously reported studies, we did also find a decrease in contralateral posterior hippocampus-DMN connectivity for the learning-decline patients, but, on the contrary, this group also displayed decreased connectivity between the pathological posterior hippocampus and the DMN. In our approach, we also found that the connectivity of the anterior hippocampus and the DAN was a major discriminant of learning-decline patients. This connectivity has been reported as crucial for encoding processes (22, 42). We hypothesized that an increased connectivity of the anterior pathological hippocampus to the DAN could relate to adaptive changes, relating to the functional adequacy of the to-be resected hippocampus. This pattern of the to-be resected hippocampus, having decreased posterior connectivity to the DMN bilaterally, but increased anterior connectivity to the DAN, could related to a higher vulnerability compared to the learning-intact, who showed the inverse pattern (see Figure 3).

Our series consists of patients with different etiologies, with a majority of patients having hippocampal sclerosis (HS). TLE is an heterogeneous disease, even in patients with HS, were heterogeneity in impairment of cognitive domains is found (43). Individual differences are common, likely related to network plasticity (44) against different etiologies and ages of presentation. In our results, different network connectivity of anterior and posterior hippocampus in patients and controls was found, likely related to the different network readjustments in our group of TLE patients, as TLE disrupts large scale cognitive networks (45). Analyzing the differences along the longitudinal axis of the hippocampus could help identify patients at risk of cognitive decline after surgery. In our series, the patients who suffered a verbal learning decline relied on increased connectivity of the anterior pathological hippocampus to the DAN, with decreased connectivity of the posterior hippocampus with the DMN. After resecting the anterior portion of the hippocampus, it is assumed that this connectivity would be disrupted, and therefore the patients suffered a verbal learning impairment.

Our study has several drawbacks. First of all, there was a small number of patients included. Second, we did not consider the extent of resection of the mesial and lateral temporal lobe, which is also associated with memory impairment after surgery (46). Also, most of our sample consists of patients with HS, which could mean that our findings are specific of this condition. Finally, we did not find significant differences between learning-intact and controls, as the discriminant function was not able to differentiate between these groups. Further studies with higher number of participants could be necessary in order to better delineate the learning-intact patients.

On the other hand, applying network analysis along the functional gradient of the hippocampal formation, we report pre-surgical differences among patients with verbal learning decline probably related to individual network plasticity. This finding supports the assumption that RSN—hippocampal connectivity is relevant for cognition, and measuring its integrity could help in the pre-surgical assessment of cognitive risks. However, several other factors should be considered, furthermost the extent of resection, pre-operative memory testing, and verbal lateralization. Nonetheless, the pre-operative connectivity of the mesial temporal area could help predict the risks of verbal learning decline after surgery for TLE. A multivariate prediction system would likely be the best approach in predicting memory deficits after surgery.

## Conclusion

Our findings support the hypothesis of an anterior-posterior functional division of the hippocampal formation and the cognitive networks. Differences in the pattern of functional connectivity of the DAN and DMN along the longitudinal axis of the hippocampus may have implications on post-operative cognitive deficits and could help identify individually which patients are more at risk of cognitive impairment. Verbal learning impairment remains an important side effect of epilepsy surgery. As a result, personalized counseling based on resting state network connectivity could help in decision making.

## Data Availability Statement

The raw data supporting the conclusions of this article will be made available by the authors, without undue reservation.

## Ethics Statement

The studies involving human participants were reviewed and approved by Clinical Research Ethics Committee of Bellvitge University Hospital. The patients/participants provided their written informed consent to participate in this study.

## Author Contributions

All authors listed have made a substantial, direct, and intellectual contribution to the work and approved it for publication.

## Conflict of Interest

The authors declare that the research was conducted in the absence of any commercial or financial relationships that could be construed as a potential conflict of interest.

## Publisher's Note

All claims expressed in this article are solely those of the authors and do not necessarily represent those of their affiliated organizations, or those of the publisher, the editors and the reviewers. Any product that may be evaluated in this article, or claim that may be made by its manufacturer, is not guaranteed or endorsed by the publisher.
